# Parahydrogen-enhanced pH measurements using [1-^13^C]bicarbonate derived from non-enzymatic decarboxylation of [1-^13^C]pyruvate-d_3_†

**DOI:** 10.1039/d4an00832d

**Published:** 2024-08-16

**Authors:** Maria Daniela Santi, Theresa Luca Katrin Hune, Gonzalo Gabriel Rodriguez, Lisa M. Fries, Ruhuai Mei, Sonja Sternkopf, Josef Elsaßer, Stefan Glöggler

**Affiliations:** a NMR Signal Enhancement Group, Max Planck Institute for Multidisciplinary Sciences Am Fassberg 11 37077 Göttingen Germany stefan.gloeggler@mpinat.mpg.de; b Center for Biostructural Imaging of Neurodegeneration, University Medical Center Göttingen Von-Siebold-Str. 3A 37075 Göttigen German

## Abstract

Alterations in pH are a hallmark in several pathologies including cancer, ischemia, and inflammation. Non-invasive magnetic resonance methods to measure pH offer a new approach for early diagnosis of diseases characterized by acid–base imbalances. The hyperpolarization with parahydrogen-induced polarization (PHIP) enhances inherently low signals in magnetic resonance experiments by several orders of magnitude and offers a suitable platform to obtain biocompatible markers in less than one minute. Here, we present an optimized preparation of an hyperpolarized H^13^CO_3_^−^/^13^CO_2_ pH sensor *via* non-enzymatic decarboxylation with H_2_O_2_ of [1-^13^C]pyruvate-d_3_ obtained by PHIP at 7 T. An improved ^13^C polarization of purified [1-^13^C]pyruvate-d_3_ in water with 36.65 ± 0.06% polarization was obtained starting from 50 mM precursor. Subsequent decarboxylation, H^13^CO_3_^−^/^13^CO_2_ exhibited 12.46 ± 0.01% of polarization at physiological pH, 45 seconds after the reaction start. Considering the dilution factor that [1-^13^C]pyruvate-d_3_ exhibits *in vivo*, we optimized our methodology to test the accuracy of the pH sensor at single digit millimolar concentration. *In vitro* pH estimations on phantoms and cell culture media demonstrated accurate pH calculations with uncertainties of less than 0.08 units. These promising results highlight the efficiency of a pH sensor generated *via* PHIP in less than one minute, with remarkable polarization, and biocompatibility suitable for future *in vivo* studies.

## Abstract 2

PHIP and non-enzymatic decarboxylation of [1-^13^C]pyruvate-d_3_ were applied to obtain, in less than one minute, [1-^13^C]bicarbonate, an accurate and biocompatible pH sensor. This probe is suitable for future *in vivo* studies of acid-base imbalances.

## Graphical abstract

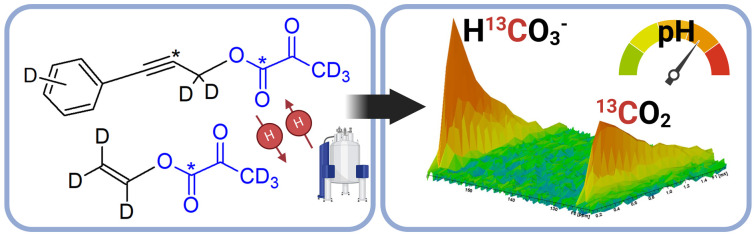

## Introduction

**Fig. 1:**
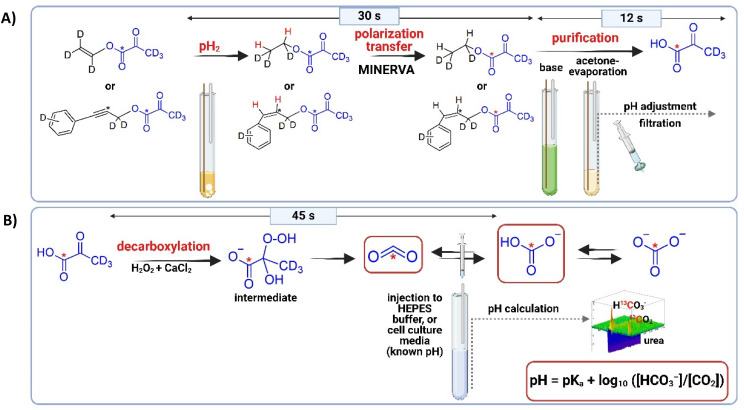
Schematic representation of the PHIP-SAH workflow; (A) full workup that includes: pH_2_ bubbling (20 s), MINERVA polarization transfer (10 s), base addition (1 s), acetone evaporation (8 s), buffer addition (1 s) and filtration (3 s); (B) decarboxylation process to obtain PHIP-H^13^CO_3_^−^/PHIP-1^3^CO_2_ (reaction takes 45 s), and pH measurement at 7 T. Own Fig. created with BioRender.com.

pH is robustly regulated in healthy tissues and is essential for physiological processes including tissue oxygenation, proper protein structure, and various biochemical reactions.^1^ However, perturbations of acid–base homeostasis are a common feature of several pathological conditions including cancer, ischemia, inflammation, renal disease, and infectious diseases.^2–4^ It is known that the acidic microenvironment generated by cancer cells correlates with aggressiveness, migration, invasion, and metastasis potential.^5–7^ All these events are associated with the Warburg effect, which involves increased glucose uptakes, anaerobic glycolysis, increased production of lactate, and an acidic extracellular pH.^8^ Moreover, it was reported that aberrant acidifications could contribute to Alzheimer's disease and Amyotrophic lateral sclerosis’ development.^9–11^ Therefore, the development of safe and rapid pH measurement may aid in the early diagnosis of acid–base imbalances and subsequent pathological disorders. Although different techniques such as magnetic resonance spectroscopy (MRS), magnetic resonance imaging (MRI), and positron emission tomography (PET) have been used to measure pH in preclinical environments, none of them was successfully translated to routine clinical use.^4,12–22^ Regarding MRS, ^31^P and ^19^F probes were used to measure pH *in vivo*. However, they present small pH dependent chemical shifts and lack of sensitivity.^13,23–27^ Concerning MRI focused techniques, several methods were developed (*e.g.* CEST and the use of Gd^3+^).^27,28^ Overall, the main reasons why the noninvasive developed probes are challenging to be clinically translatable are centered on, (i) the potential toxicity of the existing probes which makes them unfeasible to be injected in routine imaging studies in patients,^2–4,29–32^ and (ii) the low signal sensitivity of the nuclear magnetic resonance techniques. For these reasons, the use of biocompatible probes combined with signal enhancement techniques offers great potential. MRS and MRI stands out as highly versatile and valuable tools for a wide array of applications including human imaging, *in vitro* and *in vivo* chemical and biochemical studies, and unraveling molecular structures. Despite its remarkable capabilities, its effectiveness is hindered by its inherent low sensitivity.^33^ However, this limitation is effectively addressed through hyperpolarized (HP) magnetic resonance. HP enhances MRS signals by over four orders of magnitude^34^ relying on the generation of isotopically enriched, non-toxic contrast agents that enable *in vitro* and *in vivo* metabolic kinetics studies in real-time and that have been used among other for patient pH imaging.^34–36^ To generate hyperpolarized ^13^C metabolites, two approaches are largely employed. Between them, dissolution Dynamic Nuclear Polarization (dDNP) is a technique that has been used for producing HP pyruvate,^37,38^ and also to produce HP bicarbonate to perform *in vitro* and *in vivo* pH measurements.^2,39–44^ However, clinical scale dDNP is expensive and it is a slow technique that requires times between ten minutes to hours to produce HP metabolites.^45,46^ Although several HP probes were successfully developed using dDNP, the needed of sophisticated and expensive equipment, as well as, the long polarization times are most likely the reasons why translation is challenging.^2,3,40–42,47–54^ An alternative approach to hyperpolarize metabolites is based on parahydrogen-induced polarization (PHIP). This technique converts the nuclear spin singlet of parahydrogen (pH_2_) into enhanced signals within seconds. One of the pH_2_-based hyperpolarization methods, known as Signal Amplification by Reversible Exchange (SABRE), operates through reversible exchange processes between a target molecule and a metal catalyst.^55^ For the classical PHIP method, an unsaturated precursor is required to which hydrogen is added. The development of PHIP-SAH (PHIP by means of the Side Arm Hydrogenation) opened up new opportunities to hyperpolarize metabolites.^46^ Thereby, the target molecule is functionalized with an unsaturated side arm, *e.g. via* esterification. The molecule with the side arm serves as an efficient precursor to which the parahydrogen is added and this approach has demonstrated the highest yielding levels of polarization with pH_2_ so far. In particular fully deuterated precursors have shown to deliver optimal polarization conditions due to the reduction of dipolar couplings to hydrogen originating from parahydrogen, prolonging *T*_1_.^56^ Overall, the unsaturated bond is hydrogenated by using pH_2_ and a catalyst, usually in organic solvents. Next, the yielded proton spin order is transferred to a target ^13^C nucleus. In our work we use the spin order transfer sequence called MINERVA (Maximizing Insensitive Nuclei Enhancement Reached *Via* parahydrogen Amplification) for this transfer.^56^ Following the magnetization transfer, the side chain is removed by hydrolysis enabled by the addition of a base. The organic solvent is removed by evaporation and after subsequent filtration, the pH is adjusted to physiological values, and finally, after around one minute, the HP ^13^C-compound is obtained.^34^ The procedures including used precursors are shown in Fig. 1A and PHIP-SAH has overall allowed to enhance important metabolites for *in vivo* studies.^34–36,46,57–64^ Thereby ^13^C-pyruvate is used as key metabolite for a non-invasive kinetic evaluation of conversions from pyruvate to lactate in real-time *in vitro* and *in vivo*.^34,56,59,65^ Even though metabolites and especially pyruvate can be hyperpolarized with parahydrogen, so far, no biocompatible parahydrogen derived pH sensor is available at sufficient polarization (>10% ^13^C polarization). The latter we are introducing in this work by performing a non-enzymatic decarboxylation in which H_2_O_2_ present a nucleophilic addition to the α-carbonyl group of pyruvate to generate an unstable intermediate, which reacts to CO_2_ that is in equilibrium with HCO_3_^−^, acetate, and water at neutral pH (Fig. 1B).^35,66^

## Materials and methods

### Chemicals

All chemicals were purchased from Sigma Aldrich, except the precursors that were synthesized according to published procedure.^35,61,67^ Please refer to the ESI† for further information (Schemes S1 and S2†).

### Hyperpolarization and non-enzymatic decarboxylation: procedure and equipment

**Fig. 2:**
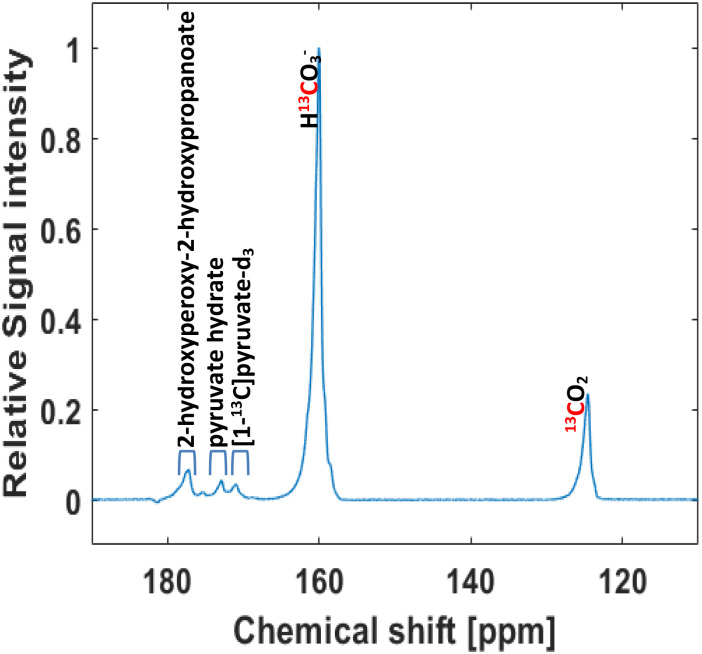
Non-enzymatic decarboxylation of HP [1-^13^C]pyruvate-d_3_ (50 mM) with H_2_O_2_ (50 mM) at 50 °C. Single ^13^C spectrum recorded at 7 T after 45 s of H_2_O_2_ injection.

[1,4-Bis(diphenylphosphino)butane](1,5-cyclooctadien)rhodium(i)-tetrafluorborat (Sigma Aldrich, catalog # 79255-71-3) was dissolved in acetone-d_6_ and used as catalyst for the PHIP procedure with a final concentration of 10 mM. After mixing with the precursor (vinyl pyruvate-d_6_ at 50 mM or phenyl propargyl pyruvate ester-d_6_ at 5.5 mM), the samples were filled into a 7-inch 5 mm NMR tube and bubbled with N_2_ for 10 seconds for degassification. *para*-enriched hydrogen was obtained at 20 K with a He-cooled parahydrogen generator provided by a Cryocooler system (Sumimoto HC-4A helium compressor, Sumimoto Cold Head CH-204 with pH_2_ reaction chamber by ColdEdge Technologies, temperature controller Lake Shore Cryotronics, Inc.) and delivered by a home-built valve and tubing system. Hyperpolarization and polarization transfer were carried out in a Bruker Avance III HD 300 narrow bore spectrometer operating at 7.05 T field strength equipped with a room temperature broadband observe (BBO) probe head for sample diameters of 5 mm (PA BBO 300S1 BBF-H-D-05 Z). Vinyl pyruvate-d_6_ was hyperpolarized by using the MINERVA sequence as described previously (detailed in the ESI, Fig. S2A and B†).^35,65^ For hyperpolarization of phenyl propargyl pyruvate ester-d_6_, a field-independent MINERVA sequence for the transfer of longitudinal spin order of parahydrogen polarization was applied as previously published (detailed in the ESI†).^36^ After finalization of both processes (30 s), 200 μL of a Na_2_CO_3_ in D_2_O (75 mM for 50 mM of the vinyl pyruvate-d_6_ precursor or 7.5 mM for 5.5 mM of the phenyl propargyl pyruvate ester-d_6_ precursor), containing EDTA (1 mM in D_2_O, which prolongs *T*_1_ and stabilize the polarization),^68^ and sodium ascorbate (50 mM in D_2O_, which decreases the polarization loss),^69^ were added to cleave the side arm and release hyperpolarized [1-^13^C]pyruvate-d_3_. Then, the NMR tube was placed outside the magnet and acetone was evaporated by passing N_2_ at 7 bar pressure through the tube, and by immersing the tube in warm water (∼79 °C) for 8 seconds.^34^ After evaporation, indicated by a reduction of the total volume by one half in these 8 s (the volume of acetone and base added were 200 μL each, so the reduction of the acetone volume was visually checked), 50 μL of HEPES (100 mM, pH 10 in D_2_O) were added to obtain physiological conditions in the final solution (1 s). The aqueous solution was then filtered from the NMR tube into a syringe by using a PVDF filter with a pore size of 1.0 μm (Fisher Scientific) to obtain in 3 s the catalyst-free signal-enhanced [1-^13^C]pyruvate-d_3_.^34^ Finally, 50 μL of H_2_O_2_ at 326 mM (final concentration 50 mM, 0.15%) was added to induce the non-enzymatic decarboxylation. 25 μL CaCl_2_ at a concentration of 3 mM was injected together with H_2_O_2_ to increase the decarboxylation rate. Fig. 1 and Fig. S1 (ESI)† show a schematic representation of the entire procedure. In Fig. 2 is shown the spectrum recorded after 45 s of the reaction.

### Monitoring of pyruvate decarboxylation

The decarboxylation reaction of pyruvate was followed spectroscopically at 50 °C using a series of 90° single scan ^1^H NMR at 7 T every 5 s, after mixing 30 mM of Sodium pyruvate in D_2_0 (concentration of pyruvate expected in the decarboxylation solution when starting from 50 mM in hyperpolarization experiments due to dilution by addition of buffer, H_2_O_2_, and CaCl_2_), with 50 mM of H_2_O_2_, and 3 mM of CaCl_2_ both in D_2_O. The integral intensities of the ^1^H signals of the methyl group of each species were converted to concentrations. Below is detailed the model described by Tickner *et al.* (2020),^70^ followed for the respective equations:
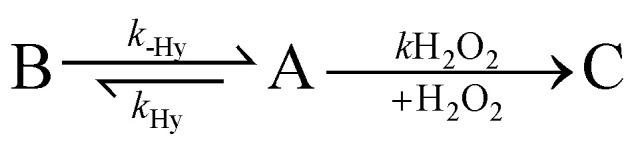
[A]_*t*+*δt*_ = [A]_*t*_ + (−*k*_H_2_O_2__[A]_*t*_[D]_*t*_ − *k*_Hy_[A]_*t*_ + *k*_-Hy_[B]_*t*_)∂*t*[B]_*t*+*δt*_ = [B]_*t*_ + (*k*_Hy_[A]*t* − *k*_-Hy_[B]_*t*_)∂*t*[C]_*t*+*δt*_ = [C]_*t*_ + (*k*_H_2_O_2__[A]_*t*_[D]_*t*_)∂*t*[D]_*t*+*δt*_ = [D]_*t*_ − (*k*_H_2_O_2__[A]_*t*_[D]_*t*_)∂*t*where A represents sodium pyruvate, B is pyruvate hydrate, C is acetate, and D is the rate of reaction between pyruvate and H_2_O_2_ obtained by fitting the integral intensities of ^1^H CH_3_ resonances of A, B, and C. *k*_-Hy_, *k*_Hy_, and *k*_H_2_O_2__ are the rates of each reaction, and in the equations *δt* is an incremental time difference.

As described by Tickner *et al.* (2020),^70^ rate constants were found by minimizing the differences between concentrations determined experimentally and the calculated values. We also maintained the same assumptions, in which species as H_2_O, pyruvate dimers or enol pyruvate, and the pathway that allows pyruvate hydrate reaction with H_2_O_2_ were not included in the model. In the ESI† are detailed the obtained kinetic parameters and the fitting (Table S1 and Fig. S3†).

### PHIP H^13^CO_3_^−^/^13^CO_2_ pH measurements in phantoms

HEPES buffer solutions in D_2_O were prepared at different pH values (6.40, 6.50, 6.61, 6.70, 6.81, 6.90, 7.20, and 7.40). The pH of each solution was adjusted using HCl (100 mM in D_2_O) and NaOH (100 mM in D_2_O), as necessary, and verified using a calibrated pH electrode. The pH measurements were performed in a Bruker Avance IV 300 7.05 T wide bore spectrometer equipped with a MicWB40 rf probe and Micro 2.5 WB gradient system at room temperature. The phantom consisted in eight empty NMR tubes labelled from 1–8 and placed surrounding a urea phantom tube, which was used as a positioning reference (Fig. 4A). 350 μL of HEPES at pH 6.4 were injected into tube 1, immediately before the injection of 175 μL of the H^13^CO_3_^−^/^13^CO_2_ (5.5 mM). After injection of H^13^CO_3_^−^/^13^CO_2_, we conducted non-localized spectroscopic experiments with low-flip angle excitations to obtain the H^13^CO_3_^−^/^13^CO_2_ integrals. Specifically, seven seconds after injection, a train of 20° flip angle pulses was initiated and a spectrum was acquired every 3 seconds. After achieving the steady state (state in which the rates of the forward and reverse reactions are equal, leading to constant concentrations of the involved species), the pH value of the respective tube was calculated by using the Henderson–Hasselbalch equation (more details in the ESI†). This procedure was repeated for each subsequent tube (2–8) to conduct the pH measurements. Each experiment was separated by a 5-minute interval to ensure that no residual signal was detected from the previous tube. This interval allowed any potential HP signal to dissipate, ensuring accurate readings. Once the pH measurements were finished for all the tubes, 500 μL of H_2_O were injected into each tube to obtain a representative ^1^H image of the setup (Fig. 4A). For this, a FLASH sequence (fast low angle shot magnetic resonance imaging) with a field of view of 30 × 30 mm^2^, a matrix size of 256 × 256, and a central slice with 3 mm thickness in the transversal plane were used. Finally, the ^1^H image and pH values obtained from the spectroscopic measurements were employed to generate a pH color-representative graph using MATLAB software (Fig. 4B).

### PHIP H^13^CO_3_^−^/^13^CO_2_ pH measurements in cell culture media

Mouse pancreatic ductal adenocarcinoma cell line (PanC02) were kindly provided by Professor Stine Pedersen (University of Copenhagen) and Professor Frauke Alves (MPI NAT, Goettingen), and they were cultured in DMEM without bicarbonate, with HEPES, supplemented with 10% Fetal bovine Serum, and 1% penicillin/streptomycin, at 37 °C, 5% CO_2_ in a humidified incubator. After four days *in vitro*, cell culture media was collected from different flasks and the pH was measured with a pH-electrode. Furthermore, fresh media-pH was also measured with the electrode. Immediately after that, we carried out pH measurement with our probe, using identical conditions as detailed before for phantoms measurements. Table S4 in the ESI† shows the individual pH values obtained for each experiment.

### Measurement of polarization and *T*_1_

Polarization values were determined from six independent experiments, by comparing the signal intensity from one hyperpolarized sample recorded with a 90° pulse to a spectrum measured at thermal equilibrium. The thermal spectrum was measured with the same acquisition parameters, *T* = 290 K using 160 scans. Peak areas from the hyperpolarized spectra were corrected according the flip angle (20°) and fitted to a decaying exponential to approximate *T*_1_ values.

### Data analysis, % polarization calculation, and statistics

The spectra were processed using TopSpin software version 4.0.8. Phase correction, line-broadening (LB 5 for EM), and baseline corrections were applied. Further analyses and 3D plot were performed using MATLAB software version R2019b. In each spectrum, the peaks of HCO_3_^−^ and CO_2_ were integrated to determine the area under the curves (AUCs). From the ratio of the AUCs, pH values were calculated by using Henderson–Hasselbalch equation. Correlation and statistical analysis were perform in GraphPad Prism version 9 software.

Polarization level was calculated using the following equation:
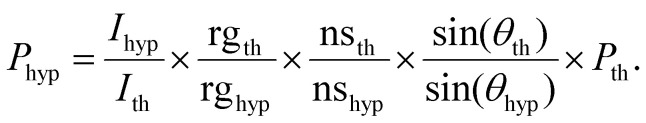


In this equation, *P*_hyp_ represents the polarization level of the sample in the hyperpolarized spectra, and *P*_th_ the polarization value of the sample in the thermal spectra. I represents the integration in the same spectra region (*e.g.*^13^C NMR signal of pyruvate at around 170 ppm) in both, hyperpolarized (*I*_hyp_) and thermal spectra (*I*_th_). rg is the receiver gain of the spectrometer, sin(*θ*_th_) = 1 and ns_hyp_ = 1, as the thermal experiments were acquired with a flip angle of 90° degrees, and a singles scan is used in hyperpolarization experiments. A comparison between one single ^13^C scan recorded for 50 mM of hyperpolarized (HP) [1-^13^C]-pyruvate-d_6_ and its corresponding thermal spectrum is showed in the ESI (Fig. S4).†

To calculate polarization levels for the H^13^CO_3_^−^/^13^CO_2_ species, we took the most conservative calculation using H^13^CO_3_^−^/^13^CO_2_ integrals after purification workup, and 45 s after H_2_O_2_ injection, in at least three different experiments.

## Results and discussion

### PHIP pH probe development

In this work, a H^13^CO_3_^−^/^13^CO_2_ probe was developed to measure extracellular pH. This probe was obtained in D_2_O at physiological conditions by non-enzymatic decarboxylation of signal enhanced 1-^13^C-pyruvate (see Fig. 1B).^35^ Fully deuterated precursors were used to restrict the effective nuclear spin system and minimizing the loss of signal enhancement.^35^ In a first step the hyperpolarization of [1-^13^C]pyruvate-d_3_ obtained from vinyl pyruvate-d_6_ was optimized and an average polarization of 36.65 ± 0.06% (see ESI†) was obtained starting from 50 mM 1-^13^C-vinyl pyruvate-d_6_ precursor. It is worth to mention that the highly obtained polarization on pyruvate would enable the preparation of the metabolic probes with liquid nitrogen cooled parahydrogen for which polarization levels would be a factor of three less but still exceed 10% ^13^C polarization. Note that this is the measured polarization of purified pyruvate in D_2_O. The purification procedure was performed by cleavage of the side arm with Na_2_CO_3_ (75 mM) supplemented with EDTA (1 mM), and sodium ascorbate (50 mM). After obtaining free [1-^13^C]pyruvate-d_3_ at 50 °C, the pH was adjusted to biocompatible conditions with HEPES-buffered D_2_O. Followed by pH adjustment, H_2_O_2_ was added to induce the non-enzymatic decarboxylation, together with CaCl_2_ to increase the decarboxylation rate.^2,71,72^ We also investigated our previously introduced Phenyl propargyl pyruvate ester precursor (PPE) at high field together with a field-independent pulsed PHIP-SAH method, which however yielded low degrees of pyruvate polarization. The complete process yielded 2.45 ± 0.01% pyruvate polarization that might be due to reduced relaxation times as compared to low field applications.^36^

We attribute that remarkable polarization value achieved on [1-^13^C]pyruvate-d_3_ starting from vinyl pyruvate-d_6_ to some changes implemented in the purification and pH stabilization procedure. In contrast to previous purification methodologies employed by us,^34–36,56,65^ that involved the use of Na_2_CO_3_ (100 mM) in H_2_O, the present methodology employed the same base, but in D_2_O, with a concentration of 75 mM, and supplemented with EDTA (which prolongs *T*_1_ and stabilize the polarization, according to dDNP experiments),^68^ and sodium ascorbate (which decreases the polarization loss during cleavage as previously reported).^69^ Notably, the evaporation procedure involved the use of N_2_ bubbling, differing from the conventional vacuum-assisted evaporation.^36^

The final adjustment of pH was performed through the addition of HEPES dissolved in D_2_O, departing from the prior use of HEPES in H_2_O. It was reported that retaining pyruvate in PBS-D_2_O, instead of PBS-H_2_O leads to lengthening of relaxation times which may play a crucial role in this process.^45^ Moreover, in a recently published study, a similar effect was observed when H^13^CO_3_^−^/^13^CO_2_ were obtained *via* hydrolysis of HP [1-^13^C]1, 2-glycerol carbonate. Hydrolysis conducted with NaOH in D_2_O affords superior polarization values and extended lifetimes compared to its counterpart in H_2_O.^52^ In this point, it is important mentioning that D_2_O is safe for *in vivo* and humans injection, as was previously informed.^45,73^ In a next step, we added H_2_O_2_ (50 mM) and found that after 45 s the reaction intermediates had sufficiently been converted into H^13^CO_3_^−^/^13^CO_2_. A total ^13^C polarization of 12.46 ± 0.01% was estimated based on the initial ^13^C thermal signal of the precursor. Fig. 2 illustrates a ^13^C spectrum recorded at 7 T using a single-90 degree pulse, post-purification, and subsequent to 45 s of H_2_O_2_ injection. H^13^CO_3_^−^/^13^CO_2_ are the main species present, together with small traces of [1-^13^C]pyruvate-d_3_ (∼171 ppm), the intermediate (2-hydroxyperoxy-2-hydroxypropanoate, ∼176 ppm), and pyruvate hydrate (∼178 ppm).^35,74^

### [1-^13^C]pyruvate-d_3_ decarboxylation kinetics

Furthermore, we monitored the sodium pyruvate decarboxylation rate following the model previously reported by Tickner *et al.*^70^ Briefly, 30 mM of sodium pyruvate (concentration expected in the decarboxylation solution when starting from 50 mM in hyperpolarization experiments) were decarboxylated by the addition of 50 mM of H_2_O_2_ in D_2_O and 3 mM of CaCl_2_ in D_2_O. The reaction was monitored spectroscopically at 50 °C using a series of 90° single scan ^1^H NMR at 7 T every 5 s. The integral intensities of pyruvate, acetate, and pyruvate hydrate were converted to concentration and plotted over time (see ESI†). By fitting these data to the kinetic model described by Tickner *et al.* using the same assumptions (*e.g.* omission of H_2_O, pyruvate dimers, or enol pyruvate species, as well as, omission of the reaction between pyruvate hydrate and H_2_O_2_),^70^ the rate of the reaction between pyruvate and H_2_O_2_ was calculated. The *k*_H_2_O_2__ value was estimated as (0.88 ± 0.14) dm^3^ mol^−1^ s^−1^ (please refer to the ESI† for further details). Even though this value demonstrate a rapid decarboxylation rate of pyruvate, and pyruvate conversion was approximately of 76%. 9 mM of uncarboxylated pyruvate (24%) were present after 45 s of H_2_O_2_ addition. Given the signal strengths shown in Fig. 2 we do not expect interferences due to the low concentration. However, if it is considered that uncarboxylated pyruvate could be transformed *in vivo* to lactate (∼182 ppm) and alanine (∼176 ppm), there are no major concerns since the residual products will not interfere with the signal of the species used for the pH measurements H^13^CO_3_^−^ (∼160 ppm), and ^13^CO_2_ (∼128 ppm), and actually could provide valuable information about real-time metabolic kinetics. Concerning H_2_O_2_ concentration, assuming a 1 : 1 relation between acetate formed and H_2_O_2_ consumed, it was observed that at 45 s the remaining concentration of H_2_O_2_ is approximately 20 mM (0.06%) in 325 μL. Human studies exist in which H_2_O_2_ in concentrations between (0.06–0.48)% were injected *via* intra-arterial or intravenous (evaluating its therapeutic potential on head and neck cancer, arteriosclerosis, wound healing) did not show toxicity.^75^ On *in vivo* experiments, the reported intravenous (median lethal dose) LD_50_ in mice was founded to be 50 000 mg per kg body weight.^76^ Therefore, given that facts, and the fact that that our solution would dilute by the blood volume (∼6 L in humans, 77–80 μL g^−1^ in mice), the residual concentration does not appear to pose a concern to humans or mice. Nevertheless, upon a potential advancement into clinical trials these procedures would need to be optimized by ideally further reducing the H_2_O_2_ content and performing a toxicology assessment.

Regarding CaCl_2_ at the final solution, this is added during decarboxylation at 3 mM, concentration that is much lower than used safely in humans and mice for calcium replenishment in arrhythmias associated with hypocalcemia, hyperkalemia, an antidote for magnesium poisoning, as co-adjuvant in hemostatic agents preparations, as well as, to resolve refractory hypotension and complete heart block, *etc*.^77–81^ Additionally, Kawai *et al.* (2011) reported the use of CaCl_2_ injected intravenously (*via* tail vein) in mice to show how this compound accelerate wound healing. In these experiments a dose of 0.6 mg per mouse was used without any observed side effects.^82^ In our injections, even if all the initial CaCl_2_ were present in the final solution, it will represent a dose of 0.0033 mg per mouse, so side effects are unlikely. Respect to rhodium residuals, we have previously reported that the levels at the final solution after filtration is (17 ± 5) μM.^56^ This concentration have not generated any side effects *in vivo* or toxicity in cells experiments, even when double injection of the probe was used.^34,56,59,65^ Finally, acetate is expected to be present in the final solution. However, it has been previously reported the use of hyperpolarized acetate as a tracer in different diseases and events (*e.g.* diabetes, cardiac and renal metabolism, glioma, *etc*.) on *in vivo* and human studies demonstrating that this compound does not pose challenges.^83–87^ In addition to that, acetate-buffers were extensively used as first-line intravenous resuscitation fluids in patients, for example during hemorrhagic shock.^88–90^

### PHIP H^13^CO_3_^−^/^13^CO_2_ validation in phantoms

During *in vivo* experiments, [1-^13^C]pyruvate-d_3_ is injected into the mouse's tail vein, thus suffering a dilution because of the mouse blood volume. Considering that the approximate blood volume of a mouse is 77–80 μL g^−1^,^91,92^ and that in our previous *in vivo* experiments with pyruvate usually C57BL6 mice with a weight of ∼20 g (10–12 weeks) are used,^34,65^ the final concentration of biomarkers when injected into the vein tail (volume injected ∼50–100 μL) will decrease about by a factor of ten.

**Fig. 3:**
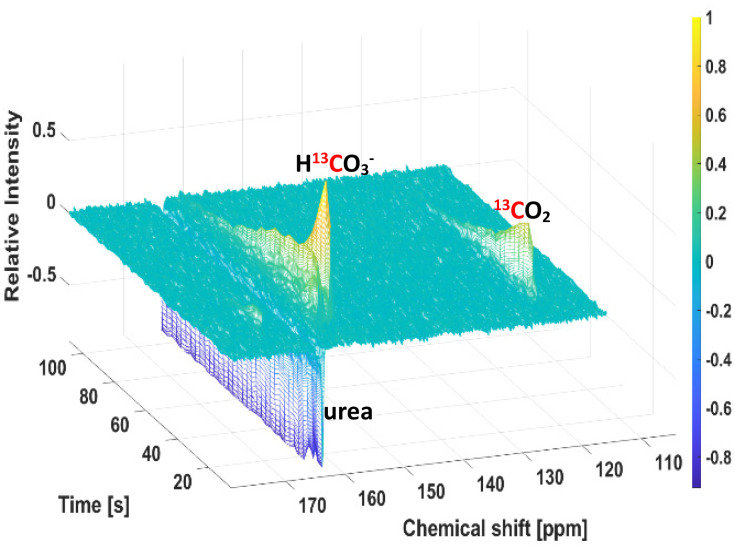
Example for a time series of ^13^C NMR spectra recorded with a flip angle of 20° upon injection of signal-enhanced H^13^CO_3_^−^/^13^CO_2_ in a phantom of pH 6.5, after non-enzymatic decarboxylation of hyperpolarized [1-^13^C]pyruvate-d_3_. A urea phantom was used in order to ensure correct position of the phantom tubes during the measurements, and its signal is in antiphase showing non-hyperpolarization.

Taking that into account, we wanted to evaluate the accuracy of the pH sensor at a 10-fold lower concentration. Following establishment of the procedure, pH measurements of eight phantoms were performed in a 7 T imaging spectrometer (more details in the ESI†). Each phantom contained HEPES buffers at different pH (6.40, 6.50, 6.61, 6.70, 6.81, 6.90, 7.20, and 7.40) in D_2_O. After filtration and decarboxylation, the solution containing H^13^CO_3_^−^/^13^CO_2_ was injected into the different tubes (one by one, and after an interval of 5 min, as explained in Materials and Methods section) containing the mentioned buffers. Upon injection, ^13^C-spectra were acquired with low-flip angle excitations (20°) to obtain H^13^CO_3_^−^/^13^CO_2_ integrals (Fig. 3 shows one example). Once achieving the steady state (state with constant concentration, according to two-way ANOVA followed by the Bonferroni's test for multiple comparisons), pH was calculated by using the Henderson–Hasselbalch equation: pH = 6.2 + log([HCO_3_^−^]/[CO_2_]),^93^ and the ratio of the H^13^CO_3_^−^/^13^CO_2_ integrals. Table 1 shows the means ± SD for H^13^CO_3_^−^/^13^CO_2_-measured pH values and their corresponding electrode-measured values, obtained after three independent experiments. Following spectroscopic measurements, the pH of the final solutions was determined with a pH electrode, and the values were in good agreement with those determined from the spectroscopic data (Table 1). This pH sensor methodology demonstrates accuracy with standard deviations lower than 0.07 pH units (expanded details in the ESI†).

**Table 1:** Measured pH by using our probe and spectroscopic data, and pH measured with an electrode

Phantom #	Electrode pH (Mean ± SD)	PHIP H^13^CO_3_^−^/^13^CO_2_ pH (Mean ± SD)
1	6.40 ± 0.01	6.37 ± 0.01
2	6.50 ± 0.01	6.50 ± 0.05
3	6.61 ± 0.01	6.60 ± 0.007
4	6.70 ± 0.01	6.65 ± 0.02
5	6.81 ± 0.01	6.76 ± 0.03
6	6.90 ± 0.01	6.91 ± 0.06
7	7.20 ± 0.01	7.15 ± 0.01
8	7.40 ± 0.01	7.35 ± 0.07

**Fig. 4:**
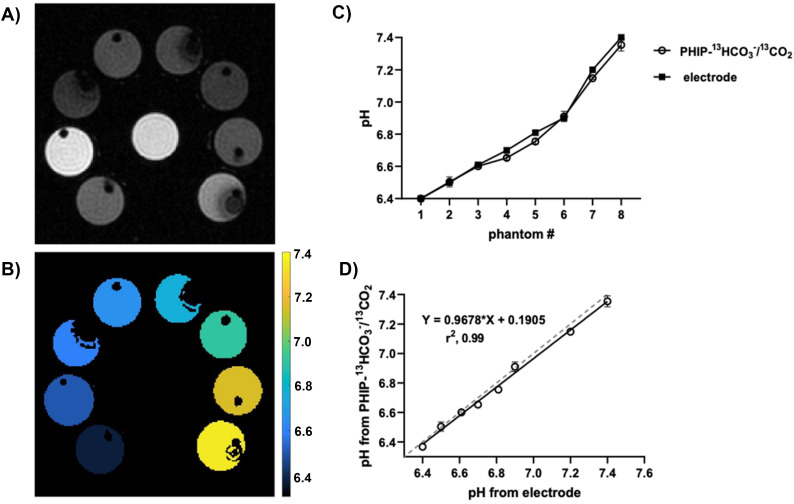
(A) Proton imaging of the setup acquired at the end of the measurements by adding H_2_O to all the measured tubes. The tube in the center corresponds to a urea phantom, which was used as a positioning reference. (B) Color graphical representation of the pH values generated in MATLAB software by using the pH values obtained spectroscopically from the ratio of hyperpolarized H^13^CO_3_^−^/^13^CO_2_ integrals, and the ^1^H image showed in A. (C) pH *vs.* phantom number (#) for PHIP H^13^CO_3_^−^/^13^CO_2_-pH and electrode-pH. No statistically differences were found after analysis by two way ANOVA followed by the Bonferroni's test for multiple comparisons using GraphPad Prism 9 software. (D) Correlation of the PHIP H^13^CO_3_^−^/^13^CO_2_-pH from spectroscopic data with the values measured using a pH electrode. A solid linear regression line with its corresponding equation is shown for the measured pH with our probe, and a dashed line that represents direct proportionality between the two variables.

Likewise, H^13^CO_3_^−^/^13^CO_2_ pH values did not differ significantly from the electrode-measured ones, according to two-way ANOVA followed by the Bonferroni's test for multiple comparisons. Fig. 4A shows a ^1^H image of our measurement setup (as explained in Materials and Methods). To ensure the correct position of the tubes, a urea-^13^C phantom was placed in the center. In (B) a color map represents the pH values obtained with our probe. (C) and (D) shows the correlation between the mean of H^13^CO_3_^−^/^13^CO_2_ pH values obtained from three independent experiments and electrode pH. The close correlation obtained reflects a rapid establishment of chemical equilibrium and equilibration of polarization between the H^13^CO_3_^−^/^13^CO_2_ species. Although previous studies reported a difference of ∼0.3 pH units where the H^13^CO_3_^−^/^13^CO_2_ pH was higher than the electrode-measured pH in the absence of the enzyme carbonic anhydrase (by using voxel by voxel estimation),^39^ we did not observe the same effect in our spectroscopic data. Our hypothesis relies on an improvement of *T*_1_,^45,52^ attributable to the generation of the signal-enhanced H^13^CO_3_^−^/^13^CO_2_ in D_2_O (instead of H_2_O).

**Fig. 5:**
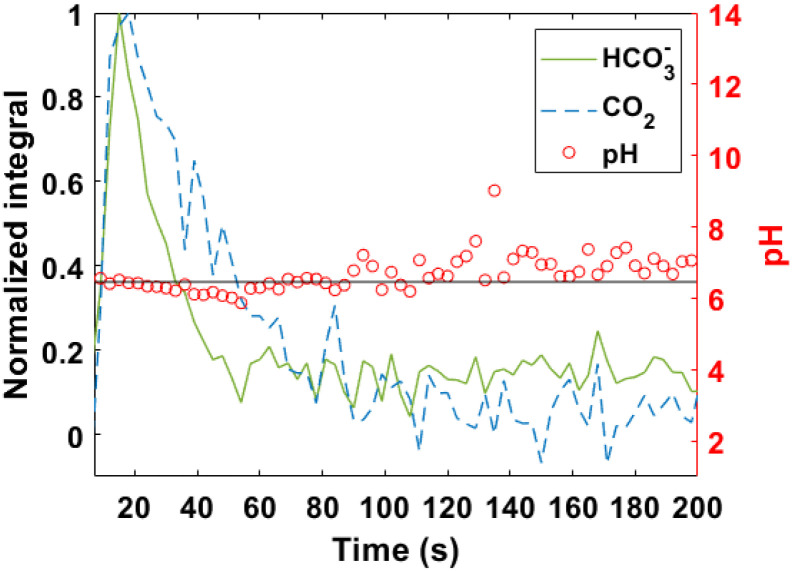
Profile of normalized integral (arbitrary unit) of H^13^CO_3_^−^ and ^13^CO_2_, and pH *vs.* time, after injection of signal-enhanced H^13^CO_3_^−^/^13^CO_2_ into a HEPES phantom of pH 6.5. pH values from the steady state were selected to calculate the ones showed in Table 1.

The *T*_1_ values for these species in D_2_O at 7 T and room temperature were (54.3 ± 9.7) s for H^13^CO_3_^−^, and (49.0 ± 3.0) s for ^13^CO_2_. This might facilitate a rapid exchange between H^13^CO_3_^−^/^13^CO_2_, and the rate of polarization loss for both species becomes similar (according to *t*-student, *p*-value from the comparison of the *T*_1_ values is 0.2, not statistically different). That rapid interconversion could contribute to maintain pH values during the steady state as is shown in Fig. 5, increasing the accuracy of the measurements.^2^ Another fact that could contribute to the improved correlation between PHIP H^13^CO_3_^−^/^13^CO_2_-pH and electrode-pH, is based on the use of an alkaline buffer (pH = 10) in our procedure to achieve physiological conditions (pH = 7), this step would be responsible for reducing CO_2_ losses prior to injection.^3^

### PHIP H^13^CO_3_^−^/^13^CO_2_ validation in cell culture media

**Fig. 6:**
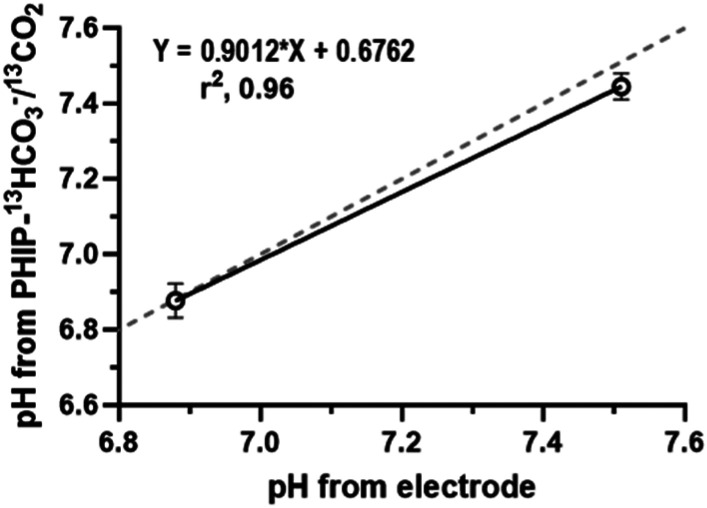
Correlation PHIP H^13^CO_3_^−^/^13^CO_2_-pH and electrode-pH values obtained from cell culture media. A solid linear regression line and equation is shown for the measured H^13^CO_3_^−^/^13^CO_2_-pH, the dashed line that represents direct proportionality between the two variables.

In order to test our probe in a more biological-complex environment, pH measurements were conducted in conditioned media from pancreatic cancer cells (PanC02 cell line) corresponding to 4 days *in vitro* (div). These measurements were compared with fresh media containing all the same supplements than the media used for PanC02 culture. Specifically, the media used was DMEM high glucose, without bicarbonate supplemented with 10% fetal bovine serum and 1% penicillin/streptomycin. Fig. 6 shows the correlation of the PHIP H^13^CO_3_^−^/^13^CO_2_-pH from spectroscopic data with the values measured using an electrode.

The mean for pH values measured with our probe are summarized in Table 2 (please see the ESI† for more details).

**Table 2:** Measured pH values in conditioned media from PanC02 cells and fresh media using our probe and spectroscopic data, and pH measured with electrodes

Media	PHIP H^13^CO_3_^−^/^13^CO_2_ pH	Electrode pH
PanC02, 4 div	6.87 ± 0.08	6.88 ± 0.01
Fresh media	7.45 ± 0.06	7.51 ± 0.01

In this case, accurate pH values were also found in agreement with those measured by using the electrode.

Lastly, we present a probe prepared by using highly polarized pyruvate (36.65%), and a non-enzymatic decarboxylation reaction to achieve the H^13^CO_3_^−^/^13^CO_2_ pH sensor, with a polarization value of 12.46%. It is important to note that the polarization losses for the H^13^CO_3_^−^/^13^CO_2_ sensor can be attributed mainly to relaxation effects (*T*_1_ 54.3 s for H^13^CO_3_^−^, and 49.0 s for ^13^CO_2_), which could be further improved in the future by optimized reactions in an automatized setup. We have validated our probe *in vitro* by measuring pH on phantoms and pancreatic cancer cell media with highly accuracy. Previous H^13^CO_3_^−^/CO_2_ studies were conducted with dDNP achieving similar levels of polarization: Ghosh *et al.* (2014), reached H^13^CO_3_^−^ with notable polarization (∼16%) by employing α-keto carboxylic acid-derivatives decarboxylation with H_2_O_2_ and validated the probe on perfused rat lungs.^2^ Besides, Mu *et al.* (2023) applied chemical reactions as hydrolysis followed by neutralization on 1,2-glycerol carbonate (GLC) to obtain H^13^CO_3_^−^ with a polarization of ∼20%, and its validation was carried out in several *in vivo* healthy and cancer mouse models.^52^ Other probes derived from synthetic compounds and amino acids were developed as HP chemical-shift based pH sensors.^47,48,51,94^ As mentioned above, dDNP is facing challenges on the development to routine clinical use. With respect to pH sensors for example, polarization times of 2 h are needed (probe obtained by direct polarization on CsH^13^CO_3_),^39^ 1.5 h (probe obtained by decarboxylation of HP α-keto carboxylic acid-derivatives),^2^ and 7 h (probe obtained by base-catalyzed hydrolysis followed by neutralization on ^13^C-GLC),^52^ together with all the time needed for reaction and post dilution steps. Therefore, parahydrogen based methods pose a scalable solution, as hyperpolarized agents can be rapidly produced on-site and even in portable devices. Finally, assessment of parahydrogen enhanced contrast agents in large volumes (40 mL as in dDNP) and patient studies are currently not feasible and are part of our ongoing work.

## Conclusions

In conclusion, we present an optimized methodology to obtain biocompatible hyperpolarized H^13^CO_3_^−^/^13^CO_2_ pH sensor within a minute using PHIP to perform pH measurements at physiological conditions with high accuracy. To achieve the probe, purified [1-^13^C]pyruvate-d_3_ with a notable ^13^C polarization of 36.65 ± 0.06% in aqueous solution and at physiological pH is decarboxylated. For this, H_2_O_2_ with an addition of CaCl_2_ yield signal-enhanced H^13^CO_3_^−^ and ^13^CO_2_ in D_2_O with an average ^13^C-polarization of 12.46 ± 0.01%. We attribute these remarkable polarization values to optimizations performed in our purification and pH adjustment procedures. We envision that the presented procedures, which offer advantages in polarization efficiency, accuracy, and speed, can be rapidly adopted for preclinical investigations and future clinical parahydrogen procedures for patient imaging.

## Author contributions

Author contributions are the following: M. D. Santi, conceptualization, data curation, formal analysis, investigation, methodology, visualization, and writing-original draft; T. L. K. Hune, investigation, formal analysis, and writing-review and editing; G. G. Rodriguez, investigation, formal analysis, and writing-review and editing; L. M. Fries, investigation, formal analysis, and writing-review and editing; R. Mei, investigation, formal analysis, and writing-review and editing; S. Sternkopf, visualization, and writing-review and editing; Josef Elaßer investigation; and S. Glöggler, conceptualization, validation, funding acquisition, resources, supervision, and writing-review and editing. All authors have given approval to the final version of the manuscript.

## Data availability

The data supporting this article have been included as part of the ESI.†

## Conflicts of interest

Stefan Glöggler is co-founder of MagniKeen

## Supplementary Material

AN-149-D4AN00832D-s001
