# Supplementary material for: Parahydrogen-enhanced pH measurements using [1-13C]bicarbonate derived from non-enzymatic decarboxylation of [1-13C]pyruvate-d3
Source: Analyst. 2024 Aug 16;149(20):5022–33. doi: 10.1039/d4an00832d (PMC11373534; doi:10.1039/d4an00832d)
Supplement: AN-149-D4AN00832D-s001 [file AN-149-D4AN00832D-s001.pdf]

Supplementary Information (SI) for Analyst.
This journal is © The Royal Society of Chemistry 2024

Supplementary Information

Parahydrogen-enhanced pH measurements using [1-^{13}C]bicarbonate derived from non-enzymatic decarboxylation of [1-^{13}C]pyruvate-d_3

Maria Daniela Santi,[a,b] Theresa Luca Katrin Hune,[a,b] Gonzalo Gabriel Rodriguez,[a,b] Lisa M. Fries,[a,b] Mei Ruhuai,[a,b] Sonja Sternkopf,[a,b] Josef Elsaßer,[a,b] and Stefan Glöggler*[a,b]

[a.] *NMR Signal Enhancement Group, Max Planck Institute for Multidisciplinary Sciences, Am Fassberg 11, 37077 Göttingen, Germany.*
[b.] *Center for Biostructural Imaging of Neurodegeneration, University Medical Center Göttingen, Von-Siebold-Str. 3A, 37075 Göttigen, German.*

Corresponding author: Dr. Stefan Glöggler, e-mail: stefan.glöggler@mpinat.mpg.de

Table of Contents

Synthesis of precursors	1
Hyperpolarization and non-enzymatic decarboxylation: procedure and equipment	1-2
Maximizing Insensitive Nuclei Enhancement Reached Via para-hydrogen Amplification (MINERVA) sequences	2
Monitoring of Pyruvate decarboxylation	2-3
PHIP $H^{13}CO_3^-$ / $^{13}CO_2$ *-pH measurements in phantoms*	3-4
PHIP $H^{13}CO_3^-$ / $^{13}CO_2$ *-pH measurements in cell culture media*	4-5
Data analysis, % polarization calculation, and statistics	5
References	5

Synthesis of precursors

The synthesis of all the precursors were published previously. Scheme S1 shows the synthesis of the phenyl propargyl pyruvate ester precursor, please refer to Korchak et al. (2018) and Jagtap et al. (2023) for more details. [1,2] Moreover, Scheme S2 shows the synthesis of vinyl pyruvate precursor (please refer to Stevanato et al. (2023) for further details).[3]

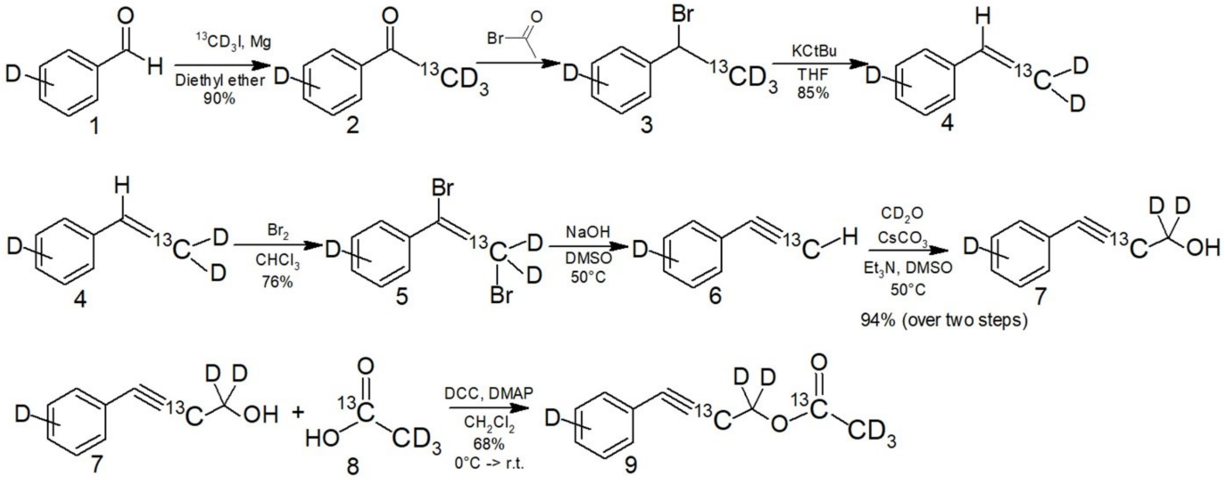

Scheme S1. Synthesis of the sidearm and pyruvate derivative, phenyl propargyl pyruvate ester precursor for PHIP-SAH.

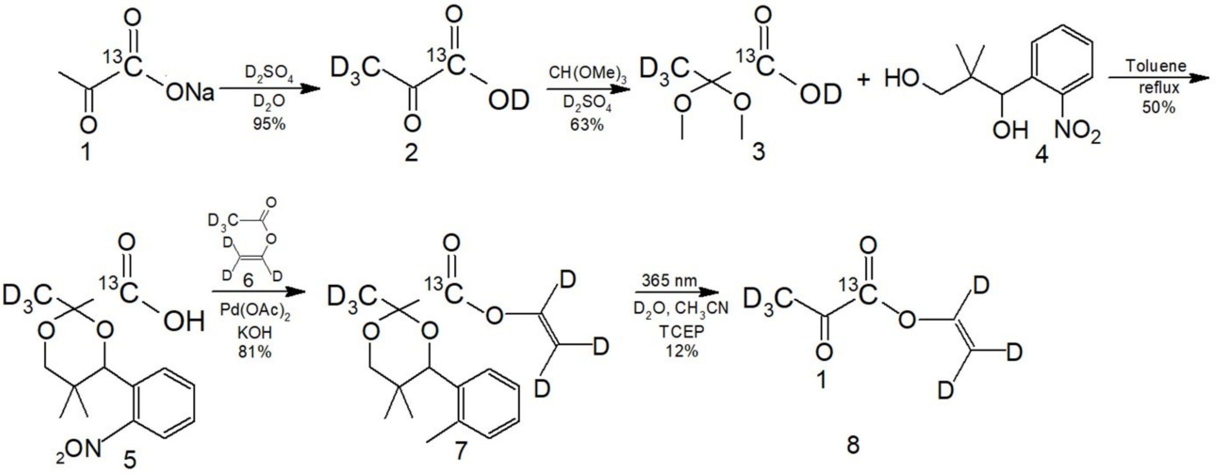

Scheme S2. Synthesis of the sidearm and pyruvate derivative, vinyl pyruvate precursor for PHIP-SAH.

Hyperpolarization and non-enzymatic decarboxylation: procedure and equipment

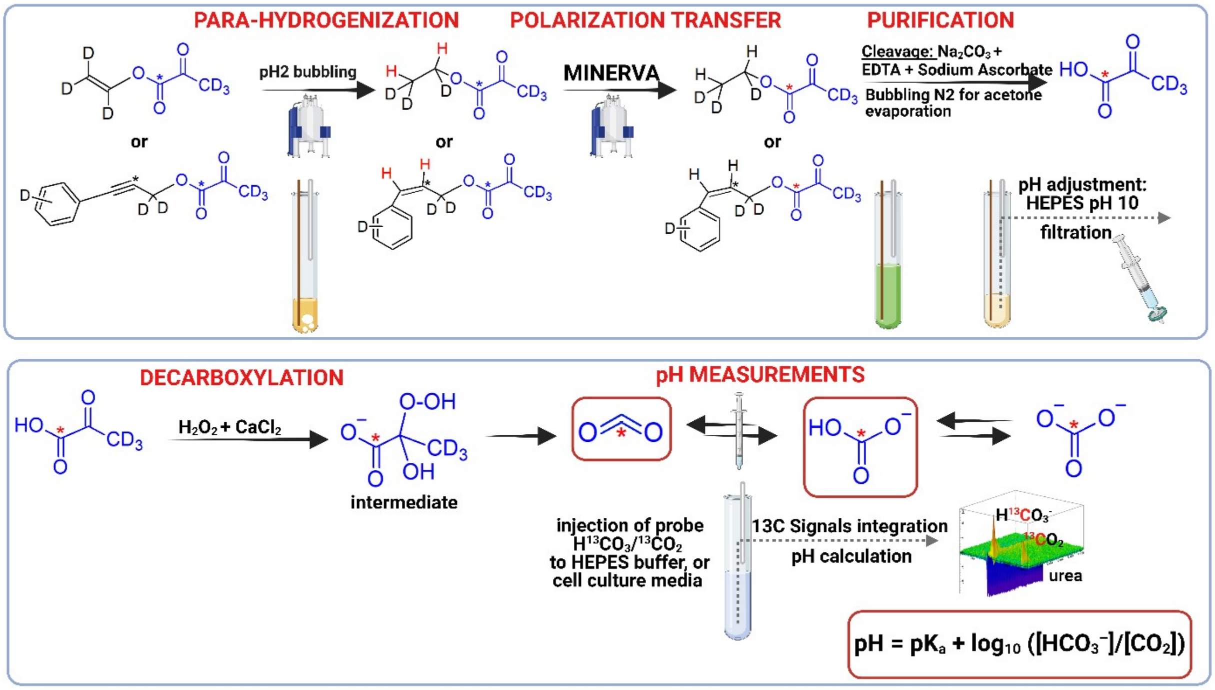

Fig. S1 Schematic representation of the PHIP-SAH workflow; A) full workup including, hydrogenation, polarization transference, cleavage and filtration (cleavage), and pH adjustment to physiological conditions; B) decarboxylation process to obtain PHIP-$H^{13}CO_3^-$ / PHIP-$^{13}CO_2$ and pH measurement at 7 T. Own Fig. created with BioRender.com.

Maximizing Insensitive Nuclei Enhancement Reached Via para-hydrogen Amplification (MINERVA) sequences

The MINERVA sequences used in this work were previously published. [5,6] Briefly, the polarization transfer relies in the J couplings. For vinyl pyruvate: J_{23}= 3.06 Hz, J_{12}= 7.1 Hz; [5] for phenyl propargyl pyruvate ester-d_6: J_{12}= 11.6 Hz, J_{13}= 0 Hz, J_{23}= 160 Hz and J_{34}= 2.3 Hz.[6] Fig. S2 A) spin order transfer and MINERVA pulse sequences applied for vinyl pyruvate-d_6 , B) spin order transfer and MINERVA pulse sequences applied for phenyl propargyl pyruvate ester-d_6. The black bars represent 90° pulses, white bars 180° pulses, and the grey bar at the phenyl propargyl pyruvate ester-d_6 sequence is used to store the heteronuclear magnetization along the applied magnetic field.

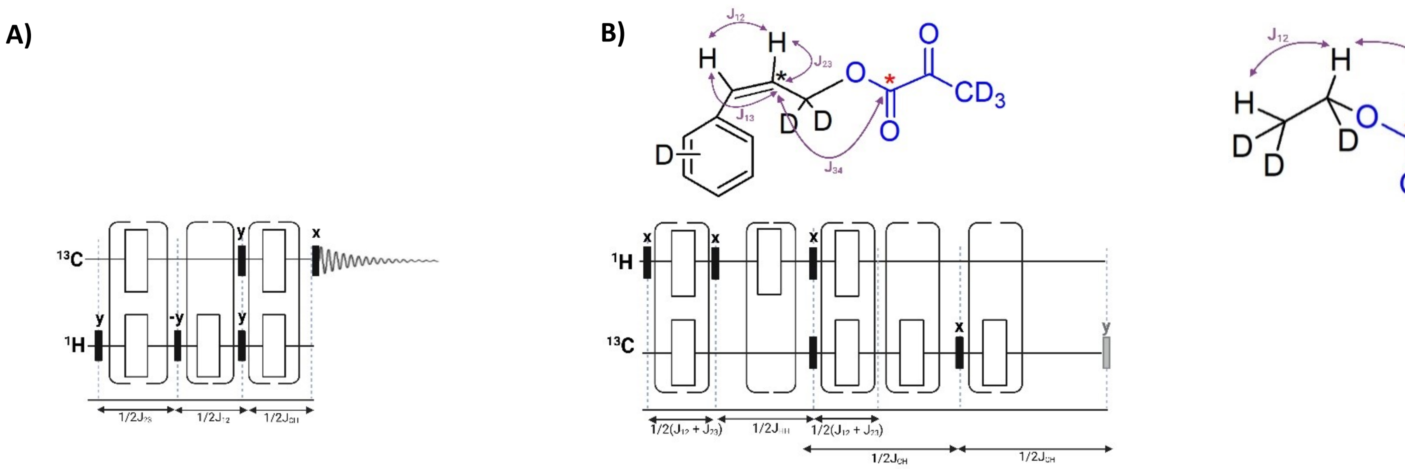

Fig. S2 A) Schematic representation of the polarization transfer; **B)** MINERVA pulse sequences applied for vinyl pyruvate-$d_{6,}$ **c)** MINERVA pulse sequences applied for phenyl propargyl pyruvate ester-$d_{6.}$

Monitoring of Pyruvate decarboxylation

Table S1 shows the parameters and Fig. S2 shows the fitting, both obtained by applying the model described by Tickner et al. (2020).[4] It is observable that for B0 (pyruvate hydrate) the model adjustment is less accurate than for the other parameters, this could be explained based on that the signal obtained from this specie along time it is very low, so SNR could be affecting the integral estimation and the fitting.

Table S1 Kinetic parameters determined from the experimental data showed in the main manuscript, according to the model described experimentally.

**Parameter**	**Mean**	**SD**
**A0**	0.0168	2.83E-04
**B0**	5.92E-04	2.10E-04
**C0**	0.0206	1.02E-04
**D0**	0.0137	0.0029
$\mathbf{k_{H202}}$	0.8808	0.1398
$\mathbf{-k_{HY}}$	0.0256	0.0118
$\mathbf{k_{HY}}$	0.8074	0.1666

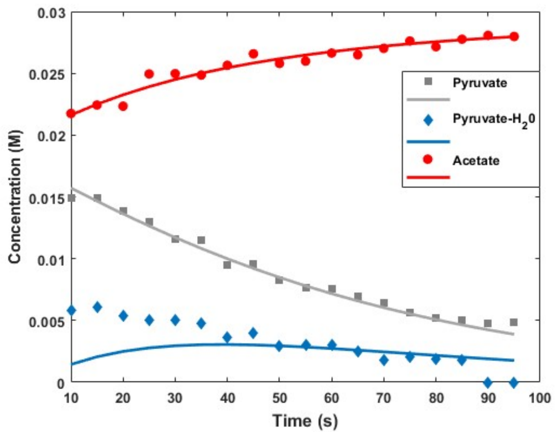

Fig. S3 Kinetics fitting of sodium pyruvate non-enzymatic decarboxylation rate with H_2O_2 (50 mM) at 50 °C, recorded at 7T every 5 s. Integral intensities were converted to concentration.

PHIP $H^{13}CO_3^-$ / $^{13}CO_2$-pH measurements in phantoms

Table S2 shows the pH values measured at the steady state of each experiment, and in Table S3 the mean of each experiment with their respective SD is summarized.

Table S2 pH values measured in HEPES phantoms at the steady state using PHIP $H^{13}CO_3^-$ /$^{13}CO_2$ in three different experiments.

Electrode pH/ PHIP $H^{13}CO_3^-$ / $^{13}CO_2$ pH	**pH 6.4**	**pH 6.5**	**pH 6.61**	**pH 6.7**	**pH 6.81**	**pH 6.90**	**pH 7.20**	**pH 7.40**
**n=1**	6.421	6.534	6.640	6.463	7.100	7.007	7.295	7.394
	6.454	6.487	6.538	6.599	6.235	7.008	7.118	7.439
	6.392	6.484	6.622	6.800	6.606	6.953	7.233	7.429
	6.179	6.486	6.639	6.802	6.405	6.816	7.129	7.410
	6.240	6.493	6.620	6.710	7.000	6.671	7.255	7.440
	6.486	6.495	6.503	6.430	6.980	6.659	6.839	7.441
**n=2**	6.240	6.340	6.593	6.755	6.791	7.185	7.190	7.294
	6.394	6.650	6.601	6.666	6.760	7.138	7.150	7.316
	6.311	6.461	6.673	6.580	6.716	6.765	7.148	7.306
	6.260	6.453	6.449	6.776	6.923	6.765	7.147	7.333

	6.336	6.216	6.428	6.684	6.718	6.791	7.133	7.360
	6.748	6.611	6.865	6.416	6.781	7.117	7.190	7.320
**n=3**	6.355	6.371	6.197	6.870	6.760	6.815	7.137	7.240
	6.348	6.797	6.209	6.679	6.677	6.923	7.122	7.414
	6.356	6.749	6.761	6.588	6.750	6.935	7.131	7.345
	6.471	6.627	6.789	6.590	6.689	6.937	7.113	7.108
	6.340	6.579	6.923	6.618	6.696	6.950	7.140	7.496
	6.270	6.250	6.770	6.727	7.010	6.945	7.173	7.256

Table S3 pH values mean and SD from PHIP $H^{13}CO_3^-$ /$^{13}CO_2$ measurements in three different experiments.

**Electrode pH**	**PHIP-$H^{13}CO_3^-$/$^{13}CO_2$**				
	**n=1**	**n=2**	**n=3**	**AVE**	**SD**
	**AVE**	**AVE**	**AVE**		
**6.40 ± 0.01**	6.362	6.383	6.356	**6.367**	**0.014**
**6.50 ± 0.01**	6.497	6.455	6.560	**6.504**	**0.053**
**6.61 ± 0.01**	6.594	6.602	6.608	**6.601**	**0.007**
**6.70 ± 0.01**	6.634	6.646	6.679	**6.653**	**0.023**
**6.81 ± 0.01**	6.721	6.781	6.764	**6.755**	**0.031**
**6.90 ± 0.01**	6.852	6.960	6.917	**6.910**	**0.055**
**7.20 ± 0.01**	7.145	7.159	7.136	**7.147**	**0.012**
**7.40 ± 0.01**	7.430	7.321	7.310	**7.354**	**0.066**

PHIP $H^{13}CO_3^-$ / $^{13}CO_2$-pH measurements in cell culture media

Table S4 shows the pH values obtained, which show being in accordance with those obtained with the electrode.

Table S4 pH values measured in PanC02 conditioned cell culture media from 4 days in vitro and fresh media at the steady state using the PHIP $H^{13}CO_3^-$ /$^{13}CO_2$ probe.

**Experiment #**	**PanC02 media**	**Average**	**Mean**	**SD**	**Experiment #**	**Fresh media**	**Average**	**Mean**	**SD**
**n=1**	6.753	6.818	**6.877**	**0.078**	**n=1**	7.523	7.453	**7.445**	**0.060**
	6.889					7.442			
	6.756					7.395			
	6.868					7.483			
	6.886					7.332			
	6.758					7.54			
**n=2**	6.722	6.827			**n=2**	7.322	7.500		
	6.862					7.568			
	6.886					7.633			
	6.823					7.385			
	6.859					7.555			
	6.810					7.535			
**n=3**	7.080	6.988			**n=3**	7.219	7.381		

	6.966					7.402			
	6.985					7.455			
	6.995					7.529			
	7.000					7.332			
	6.899					7.351			
**n=4**	6.782	6.875							
	6.790								
	6.889								
	6.927								
	6.999								
	6.863								

Data analysis, % polarization calculation, and statistics

Fig. S4 shows the comparison between one single ^{13}C scan recorded for 50 mM of hyperpolarized (HP) [1-^{13}C]-pyruvate-d_6 and its corresponding thermal spectrum.

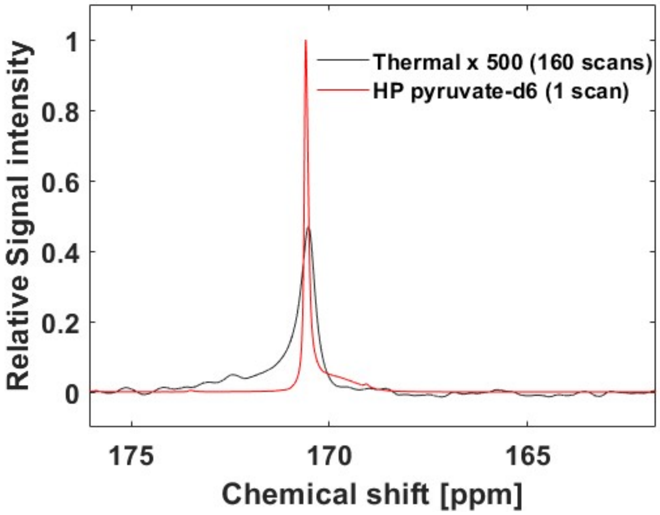

Fig. S4 Overlay of the hyperpolarized spectrum (single scan) of [1-^{13}C]vinyl pyruvate-d_6 (red) immediately after purification process (cleavage, evaporation, buffer addition, and filtration), and the thermal spectrum of the same sample (black) with 160 averages and scaled up by a factor of 500. The average ^{13}C polarization is (36.62 ± 0.06) %.

To calculate polarization levels for the $H^{13}CO_3^-$ / $^{13}CO_2$ species, we took the most conservative calculation using $H^{13}CO_3^-$ / $^{13}CO_2$ integrals after purification workup, and 45 s after H_2O_2 injection, in at least three different experiments.

References

1 S. Korchak, S. Yang, S. Mamone and S. Gloggler, *ChemistryOpen*, 2018, **7**, 344-348

2 A. P. Jagtap, S. Mamone, S. Gloggler, *Magn Reson Chem* 2023, **61**, 674-680.

3 G. Stevanato, Y. Ding, S. Mamone, A. P. Jagtap, S. Korchak, S. Gloggler, *J Am Chem Soc* 2023, **145**, 5864-5871.S.

4 B. J. Tickner, P. J. Rayner and S. B. Duckett, *Anal Chem*, 2020, **92**, 9095-9103.

5 T. Hune, S. Mamone, H. Schroeder, A. P. Jagtap, S. Sternkopf, G. Stevanato, S. Korchak, C. Fokken, C. A. Muller, A. B. Schmidt, D. Becker and S. Gloggler, *Chemphyschem*, 2023, **24**, e202200615.

6 S. Mamone, A. P. Jagtap, S. Korchak, Y. Ding, S. Sternkopf and S. Gloggler, *Angew Chem Int Ed Engl*, 2022, **61**, e202206298.
